# Glycoprotein folding and quality-control mechanisms in protein-folding diseases

**DOI:** 10.1242/dmm.014589

**Published:** 2014-03

**Authors:** Sean P. Ferris, Vamsi K. Kodali, Randal J. Kaufman

**Affiliations:** 1 Department of Biological Chemistry and Medical Scientist Training Program, University of Michigan, Ann Arbor, MI 48109, USA; 2 Center for Neuroscience, Aging and Stem Cell Research, Sanford-Burnham Medical Research Institute, 10901 N. Torrey Pines Road, La Jolla, CA 92037, USA

**Keywords:** N-glycosylation, Glycoprotein folding, ER quality control, ER-associated degradation, ER export

## Abstract

Biosynthesis of proteins – from translation to folding to export – encompasses a complex set of events that are exquisitely regulated and scrutinized to ensure the functional quality of the end products. Cells have evolved to capitalize on multiple post-translational modifications in addition to primary structure to indicate the folding status of nascent polypeptides to the chaperones and other proteins that assist in their folding and export. These modifications can also, in the case of irreversibly misfolded candidates, signal the need for dislocation and degradation. The current Review focuses on the glycoprotein quality-control (GQC) system that utilizes protein N-glycosylation and N-glycan trimming to direct nascent glycopolypeptides through the folding, export and dislocation pathways in the endoplasmic reticulum (ER). A diverse set of pathological conditions rooted in defective as well as over-vigilant ER quality-control systems have been identified, underlining its importance in human health and disease. We describe the GQC pathways and highlight disease and animal models that have been instrumental in clarifying our current understanding of these processes.

## Introduction

Over the past 30 years, the pathogenesis of multiple human genetic disorders has been directly linked to the retention of misfolded proteins in the endoplasmic reticulum (ER), sometimes driven by mutations as modest as a single amino acid substitution (Welch, 2004). These diseases – α1-antitrypsin (α1AT) deficiency, cystic fibrosis, combined factor V and VIII deficiency (F5F8D), to name a few – have been termed ER storage diseases (Kim and Arvan, 1998; Rutishauser and Spiess, 2002; Schröder and Kaufman, 2005). Interestingly, some of these mutant proteins still possess the biological activity of their wild-type counterparts (Drumm et al., 1991; Bhargava et al., 2012), suggesting that improving the exit of the mutant protein from the ER might reduce disease pathology. The mechanism(s) by which proteins with a native conformation selectively transit the ER to the Golgi apparatus, whereas misfolded proteins are retained in the ER and/or are degraded, is termed ER quality control (ERQC) (de Silva et al., 1990; Araki and Nagata, 2011). Such a failsafe mechanism ensures that the assembly line of nascent proteins in the ER does not export defective material. Quality control occurs at every stage of protein biosynthesis in the ER – during co-translational translocation, post-translational modification, chaperone-assisted folding, assembly of multi-subunit complexes, trafficking and export – with mechanisms in place for immediate recognition of mutant and/or misfolded proteins for degradation. This Review focuses on one aspect of ERQC that utilizes N-glycosylation to ascertain the ‘foldedness’ of the ER itinerant, which we refer to as glycoprotein quality control (GQC). In the first part, we describe the process of co-translational glycosylation of nascent polypeptides and the mechanism of GQC, including the role of N-glycan modification in assisting the folding, export and degradation of nascent glycopolypeptides. We then highlight how studies into the molecular mechanisms underlying GQC have provided insights into human diseases caused by defects in this pathway. In some cases, these insights could pave the way for therapeutic interventions that could alleviate disease.

## Stages in protein N-glycosylation

Glycosylation has been described as the most common post-translational modification of proteins (Apweiler et al., 1999), with the majority of proteins produced in the ER bearing covalent attachment of an oligosaccharide to asparagine (Asn) side-chains (N-linked glycosylation). These oligosaccharides, termed N-linked glycans or simply N-glycans, are added to proteins in the ER, where they serve as an entry pass to an intricate glycoprotein-specific portion of the ERQC system – GQC – that ultimately enhances the export efficiency of high-quality glycoprotein products (Roitsch and Lehle, 1989).

**Fig. 1.**
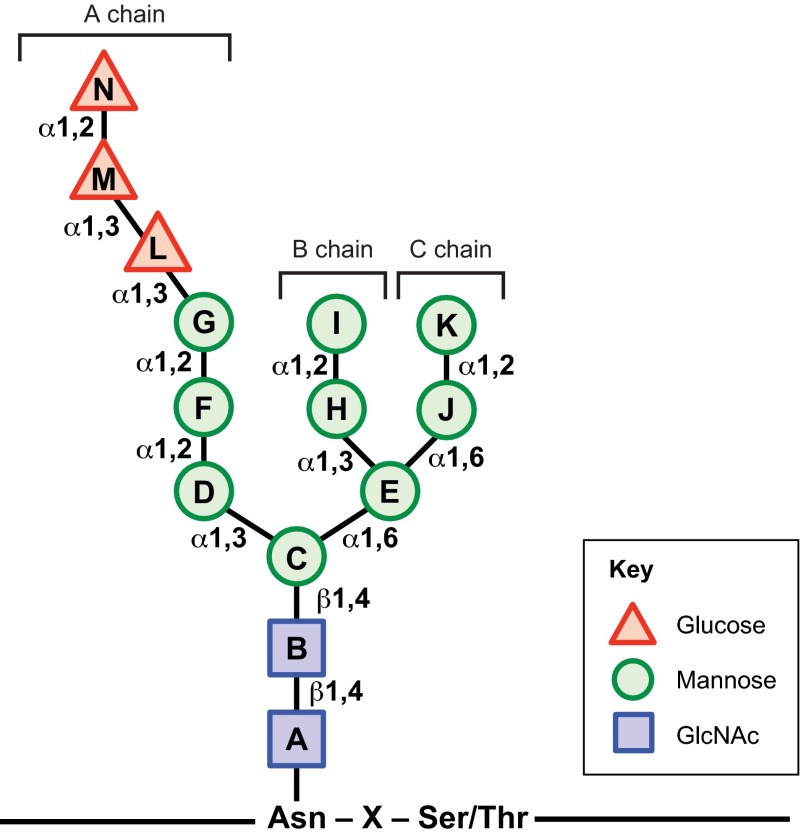
**Structure of the N-linked core glycan**. The triantennary tetradecaoligosaccharide is assembled on the ER membrane and is covalently linked to the Asn side chains in the context of the N-glycosylation sequon of newly translocated proteins. The 14-sugar form, starting from the Asn residue, contains two N-acetylglucosamine (GlcNAc, squares), nine mannose (circles) and three glucose (triangles) residues. The three branches – A, B and C – are illustrated. Glycosidic linkage types are indicated next to the connectors.

Protein N-glycosylation occurs in two stages: (1) assembly of a glycan molecule and (2) transfer of the glycan onto a nascent protein. In stage 1, a tetradecaoligosaccharide consisting of three glucose, nine mannose and two N-acetylglucosamine residues (Glc_3_Man_9_GlcNAc_2_) is assembled on an ER-membrane-anchored lipid called dolichol phosphate (Dol-P). Multiple glycosyltransferases utilizing specific sugar donors coordinate stepwise sequential assembly resulting in a dolichol-pyrophosphate-linked triantennary core oligosaccharide (Fig. 1) that is suitable for transfer to appropriate polypeptide substrates (Larkin and Imperiali, 2011; Schwarz and Aebi, 2011). The biosynthesis of the 14-sugar N-glycan precursor core is initiated on the cytoplasmic face of the ER membrane. The initial transfer of GlcNAc-P from uridine diphosphate (UDP)-GlcNAc to a Dol-P, generating Dol-P-P-GlcNAc, is catalyzed by GlcNAc-1-phosphotransferase. The nucleoside antibiotic tunicamycin specifically inhibits this enzyme, resulting in the accumulation of unglycosylated, misfolded proteins in the ER. Subsequent steps involve the addition of a second GlcNAc residue followed by five mannose residues using UDP-GlcNAc and GDP-Man donors, respectively. During this process, the ER-membrane-anchored heptasaccharide (Man_5_GlcNAc_2_) is ‘flipped’ across the ER membrane from the cytosolic side to the ER luminal side to allow assembly of the remainder of the glycan on the luminal surface (Sanyal and Menon, 2009). Inside the ER lumen, four mannose residues and three glucose residues are sequentially transferred onto the glycan using Dol-P-Man and Dol-P-Glc as donors, respectively, generating a Glc_3_Man_9_GlcNAc_2_-P-P-Dol tetradecaoligosaccharide.

In the second stage of N-glycosylation, the membrane-bound multi-subunit complex oligosaccharyltransferase (OST) associates with the translocon pore on the ER membrane and catalyzes the covalent linkage of the 14-sugar N-glycan to the Asn amide group of N-glycosylation sequons (Asn-X-Ser/Thr, where X can be any amino acid except proline) as they emerge into the lumen (Mohorko et al., 2011). This reaction generates Dol-P-P as a by-product, which is recycled for use in glycan assembly (stage 1).

## Glycoprotein folding

A recurring theme in GQC (and protein quality control in general) is the close association of the nascent glycoprotein not only with chaperones that assist in its proper folding, but also with those that target it for degradation. This arrangement, despite being overzealous in certain instances, ensures that misfolded, aggregation-prone glycoproteins are readily degraded. For GQC, sequential cleavage of sugar residues from the N-glycans is a major factor in determining the fate of ER glycoproteins. One major question in cell biology for the last 25 years is why the cell expends energy to assemble the complex N-glycan core, only to then catalyze its sequential trimming. Studies over recent years suggest that the addition and modification of the N-glycan core is intimately linked to the folding, disulphide-bond formation and complex assembly of glycoproteins.

The α1-2 glycosidic linkage between the outermost glucose residues (‘M’–‘N’, Fig. 1) from the N-glycan of the nascent glycoproteins is cleaved almost immediately by the membrane-bound enzyme α-glucosidase I (GS-I), forming Glc_2_Man_9_GlcNAc_2_ (Hubbard and Robbins, 1979). This step prevents re-binding of the processed N-glycan by OST (Helenius and Aebi, 2004) and promotes binding to a recently discovered membrane-anchored ER protein, called malectin (Schallus et al., 2008). Based on studies using the model substrates α1AT and hemagglutinin, malectin was found to preferentially bind misfolded proteins, preventing further progress along the folding pathway and directing them to the ER-associated degradation (ERAD) pathway (Chen et al., 2011; Galli et al., 2011).

**Fig. 2.**
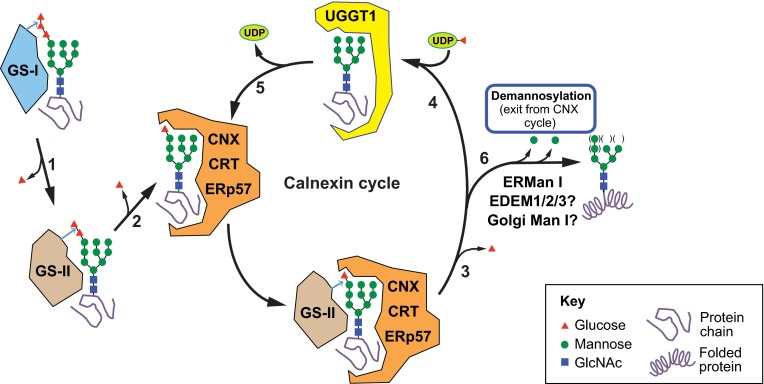
**N-glycan processing in the CNX cycle**. Glycoproteins first enter the CNX cycle after the two terminal glucose residues (red triangles) of the attached N-glycan are cleaved by glucosidases I and II (GS-I and GS-II; steps 1 and 2). The resulting monoglucosylated N-glycan binds to the lectin-like chaperones CNX and CRT. The substrate dissociates from CNX/CRT upon GS-II-mediated removal of the terminal glucose residue from the N-glycan (step 3). At this point, the glycoprotein substrate’s folding status is surveyed by the ‘folding sensor’ component of the CNX cycle, UGGT1, which specifically binds nearly-native folding forms (step 4) and reglucosylates them. Reglucosylated substrates bind to CNX/CRT once again and re-enter the CNX cycle (step 5). Substrates eventually exit the CNX cycle upon demannosylation (removal of mannose residues; green circles) of N-glycans (step 6). The mechanism for permanent exit from the cycle involves either termination of UGGT1 reglucosylation activity of demannosylated N-glycans, or active recognition of demannosylated forms by ER exit machinery or ERAD components.

Once the glycoprotein passes the malectin GQC checkpoint, a multi-subunit α-glucosidase – GS-II – removes the second glucose residue (‘M’, Fig. 1), generating Glc_1_Man_9_GlcNAc_2_ glycans that have affinities in the submicromolar range for ER lectin-like chaperones calnexin (CNX) and calreticulin (CRT) (Michalak et al., 2002; Rutkevich and Williams, 2011; Wijeyesakere et al., 2013). Glycoprotein association with CNX or CRT marks the beginning of the ‘calnexin cycle’ (Hammond et al., 1994) (Fig. 2). CNX and CRT are thought to associate in similar ways with Glc_1_Man_9_GlcNAc_2_-glycoproteins, but CNX is a membrane-anchored protein and CRT is a luminal homolog. Both proteins consist of: (1) a globular domain that harbors both the oligosaccharide- and calcium-binding sites; and (2) an elongated arm, also known as the P domain because of its proline-rich sequence motifs (Ellgaard et al., 2001; Schrag et al., 2001; Michalak et al., 2009; Kozlov et al., 2010a; Wang et al., 2012). Although the oligosaccharide-binding site in the globular domain of CRT has been well established (Kozlov et al., 2010a), how polypeptides might bind CNX and CRT is less clear. Recent structural studies have identified possible peptide-binding sites in both the lectin and arm domains of CRT (Chouquet et al., 2011; Pocanschi et al., 2011). The P domain binds to a luminal thiol-disulfide oxidoreductase, ERp57 (Frickel et al., 2002; Kozlov et al., 2006), and a peptidyl prolyl isomerase, cyclophilin B (CypB) (Kozlov et al., 2010b). Together, CNX or CRT (CNX/CRT), ERp57 and CypB assist the nascent glycoprotein in achieving a native conformation and correct disulfide pairings.

A second deglucosylation (removing residue ‘L’, Fig. 1) by GS-II prevents rebinding of the glycoprotein to the CNX/CRT-ERp57 complex (D’Alessio et al., 2010). At this stage, another key component of the CNX cycle springs into action: UDP-Glc:glycoprotein glucosyltransferase 1 (UGGT1) binds to fully deglucosylated N-glycans and ascertains whether the substrate has achieved the native conformation. Completely folded glycoproteins are promptly released by UGGT1 and are allowed to proceed along the ER-to-Golgi anterograde trafficking pathway. In contrast, substrate glycoproteins with non-native folds selectively bind to UGGT1 via solvent-exposed hydrophobic patches in conjunction with the deglucosylated N-glycan, and undergo reglucosylation using UDP-Glc as a Glc donor. This results in glycoproteins with monoglucosylated N-glycans that can once again enter the CNX cycle for folding assistance.

The ability of UGGT1 to discriminate folded from misfolded substrates is crucial for GQC. Studies using purified UGGT1 suggest the molecular mechanism by which UGGT1 recognizes its glycoprotein substrates is bipartite (Trombetta and Parodi, 1992; Sousa and Parodi, 1995). For glucosylation, the substrates must have at a minimum the innermost GlcNAc residue of the N-glycan, and harbor solvent-exposed hydrophobic residues (Sousa and Parodi, 1995). Using artificially constructed heterodimers of folded and misfolded ribonuclease monomers, UGGT1 was found to reglucosylate N-glycans only on the misfolded half (Ritter and Helenius, 2000), suggesting that, for UGGT1-mediated reglucosylation, the N-glycan must be near the misfolded region of the substrate. Interestingly, for a different glycoprotein substrate, UGGT1 was reported to reglucosylate N-glycans that were more distant (40 Å) from local hydrophobic regions (Taylor et al., 2004). Despite these substrate-specific differences, it seems that UGGT1 can survey glycoprotein substrates for misfolded regions and reglucosylate attached N-glycans when appropriate (Fig. 2). Reglucosylation mediated by UGGT1 can promote association with CNX/CRT-ERp57 and the thiol-disulfide isomerase activity of ERp57 can promote proper disulfide bond formation (Zapun et al., 1998).

In addition to the CNX cycle, glycoprotein itinerants are serviced by the ER-resident molecular chaperone BiP. A member of the Hsp70 family of proteins, BiP is the most abundant chaperone in the ER, and consists of an N-terminal nucleotide-binding domain (NBD) and a C-terminal substrate-binding domain (SBD). BiP is a peptide-dependent ATPase that can either increase or decrease the folding rate of protein ligands (Bukau et al., 2006). ATP-bound BiP binds to hydrophobic patches in nascent polypeptides that get ‘locked’ into the SBD upon hydrolysis of ATP to ADP, a process that is accelerated by an ER co-chaperone of the DnaJ family (a so-called ERdj protein). Consequently, BiP ‘holds’ the substrate, allowing it to attain its native conformation, and then permits the substrate to assemble with other subunits, as well as promotes accessibility to other chaperones such as the protein disulfide isomerase (PDI) family members that generate and rearrange disulfide bonds that are properly paired. Seven ERdj proteins (ERdj1-ERdj7) have been characterized so far, with functions ranging from ensuring nascent polypeptides are serviced by BiP immediately after entering the ER, to targeting terminally misfolded proteins for degradation (Otero et al., 2010). The substrate protein is released from BiP upon exchange of ADP with ATP, triggered by the guanine nucleotide exchange factor BAP (BiP associated protein) (Chung et al., 2002; Bukau et al., 2006).

## Glycoprotein export

How does the substrate protein escape the CNX cycle and the BiP-assisted cycles of protein folding? Once again, N-glycan trimming plays a crucial role in this process (Caramelo and Parodi, 2008). If the substrate protein does not attain its native conformation after repeated interactions with CNX/CRT, BiP or other chaperones of the ER, it is targeted for degradation by the ERAD pathway, described below. By contrast, if the substrate protein assumes a functionally competent three-dimensional structure, it traffics to the Golgi apparatus en route to its final destination. For forward transport of secretory proteins from the ER to the Golgi, two mutually nonexclusive mechanisms have been proposed: (1) bulk-flow and (2) receptor-mediated transport (Warren and Mellman, 1999).

Glycoproteins entering the Golgi through bulk flow are immediately demannosylated by one or more of the resident mannosidases: Golgi Man I (A, B, C) and Golgi Man II (Moremen, 2002). These Golgi mannosidases can remove mannoses from Glc_0–3_Man_8–9_GlcNAc_2_ N-glycans, allowing processing of all glycoproteins arriving at the Golgi, including the ones that enter owing to abnormal or failed ER quality control (Gabel and Bergmann, 1985; Moremen, 2002). Natively folded, demannosylated substrates are glycosylated and trafficked to their final destination, whereas misfolded glycoproteins are recognized by quality-control systems that operate in the Golgi complex, and are delivered to lysosomes for degradation (Arvan et al., 2002).

Receptor-mediated trafficking in the ER utilizes N-linked glycans. For example, ERGIC-53 (along with VIPL and VIP36) is thought to act as a cargo receptor, transferring correctly folded substrates to COP-II vesicles for transport out of the ER to the Golgi (Barlowe, 2003; Kamiya et al., 2008; Kamiya et al., 2012). Similarly to UGGT1, ERGIC-53 might have a bipartite recognition signal and the ability to sense folding status of its substrates (Appenzeller-Herzog et al., 2005). As an example, ERGIC-53 recognizes both the high-mannose type N-glycan and a β-hairpin loop structure present only in correctly folded cathepsin Z, an ERGIC-53 binding substrate (Appenzeller-Herzog et al., 2005). Significantly, the autosomal recessive bleeding disorder F5F8D is caused in two-thirds of patients by complete loss-of-function mutations in *LMAN1*, the gene encoding ERGIC-53 (Nichols et al., 1998). The other third of patients have mutations in the multiple clotting factor deficiency 2 (*MCFD2*) gene, which encodes a calcium-binding protein that interacts with ERGIC-53 to form a heterodimer required for trafficking of factors V and VIII from the ER to the Golgi (Zhang et al., 2003). F5F8D (described in more detail below) was the first human genetic disease identified that results from defective trafficking of proteins out of the ER.

## Degradation of misfolded glycoproteins

### ER-associated degradation (ERAD)

**Fig. 3.**
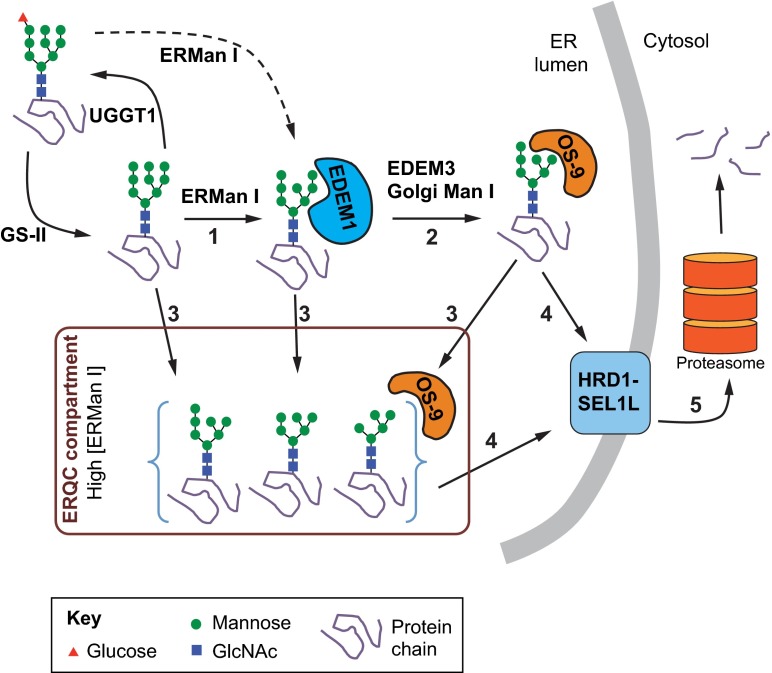
**Glycoprotein ERAD**. Degradation of terminally misfolded glycoproteins through ERAD is probably initiated by cleavage of the terminal B-chain mannose (green circles) of Man_9_GlcNAc_2_ (and possibly Glc_1_Man_9_GlcNAc_2_) N-glycan forms by ER mannosidase I (ERManI, step 1). This results in the formation and recognition of this specific Man_8_GlcNAc_2_ form by ER-degradation-enhancing alpha-mannosidase-like 1 (EDEM1). Then, removal of the terminal C-chain mannose, either: (1) directly by EDEM3 or possibly Golgi mannosidase I (step 2), or (2) by highly concentrated ERMan I in the ERQC compartment (step 3), exposes an α1-6 linked mannose that is recognized by OS-9. OS-9 facilitates transport of the misfolded substrate to the core ERAD HRD1-SEL1L complex (step 4), and subsequent retrotranslocation to the cytoplasm for degradation by the proteasome (step 5).

Trimming of mannose residues from N-linked glycans predicates productive transport of one subset of glycoproteins through the Golgi complex and another subset – typically irreversibly misfolded glycoproteins – to sequestration inside the ER and degradation by ERAD (Aebi et al., 2010; Hebert and Molinari, 2012; Olzmann et al., 2013) (Fig. 3). ERAD is a process in which misfolded proteins are retrotranslocated from the ER to the cytosol and subsequently degraded by the ubiquitin-proteasome system.

The archetypal enzyme that catalyzes the removal of the terminal mannose from the B chain of the N-glycan (residue ‘I’, Fig. 1) and initiates the events of export and/or ERAD is ER mannosidase I (ERMan I) (Gonzalez et al., 1999; Tremblay and Herscovics, 1999). Glycoproteins that are subject to degradation are further demannosylated by one or more of the following: (1) the mannosidase activity of the ER-degradation-enhancing mannosidase-like proteins (EDEM1, 2 or 3) (Kanehara et al., 2007; Olivari and Molinari, 2007); (2) multiple rounds of demannosylation by ERMan I (Hosokawa et al., 2003; Wu et al., 2003); or (3) the action of Golgi mannosidases during ER-to-Golgi cycling (Hosokawa et al., 2007; Kukushkin et al., 2011). Crucially, removal of the mannose residue ‘G’ (Fig. 1) precludes the substrate from UGGT1-mediated re-glucosylation and re-entry into the CNX cycle, ending any further attempts at protein folding. Removal of the terminal mannose residues from the B and C chains (residues ‘I’ and ‘K’, Fig. 1; Fig. 3), by contrast, allows binding of the N-glycan by the ERAD lectins OS-9 and XTP3-B, promoting degradation. OS-9 and XTP3-B are luminal proteins containing mannose-6-phosphate receptor homology (MRH) domains that specifically bind to terminal α-1,6-mannose residues to facilitate transfer of terminally misfolded glycoproteins to the membrane-associated ERAD complex for retrotranslocation and degradation (Kanehara et al., 2007; Christianson et al., 2008; Hosokawa et al., 2010).

Multiple protein complexes that are resident on the ER membrane have been proposed to be the sites of retrotranslocation of ERAD substrates and eventual entry into the ubiquitin proteasome pathway (Hoseki et al., 2010; Hampton and Sommer, 2012). One such complex is the HRD1-SEL1L ERAD complex (Fig. 3). HRD1 is an ER-membrane-localized E3 ubiquitin ligase that polyubiquitylates ERAD substrates on the cytoplasmic face of the ER membrane. SEL1L is a membrane-associated glycoprotein that interacts with an assortment of ERAD regulators, including OS-9, XTP3-B, EDEM1 and EDEM3, which facilitate substrate transfer from the protein-folding components to the ERAD complex (Mueller et al., 2008). An active area of investigation is the molecular definition of the adaptor proteins, their interactions and the mechanism for delivery to the ERAD complex (Araki and Nagata, 2011). Given that OS-9 and XTP3-B can bind to misfolded polypeptides devoid of any N-glycans, the MRH domains of OS-9 and XTP3-B might act to facilitate association with SEL1L through interaction with its N-glycans.

## Degradation of ER protein complexes and aggregates

**Fig. 4.**
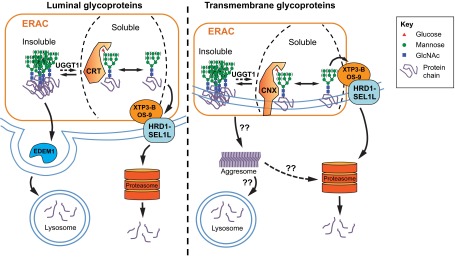
**Degradation of insoluble and soluble ER glycoproteins**. Some misfolded glycoproteins form insoluble aggregates or ordered polymers in the ER, and UGGT1-mediated modification of the glucosylation status (monoglucosylated or unglucosylated) might play a role in limiting insolubility. The soluble-to-insoluble transition might occur via compartmentalization in the ERAC, and insoluble substrates might also be resolubilized. Soluble forms of both luminal and transmembrane glycoproteins tend to be degraded through ERAD, whereas insoluble forms tend to be degraded via autophagy, although the mechanism by which insoluble ER proteins get to the lysosome is not entirely clear. Luminal insoluble glycoproteins might be packaged into EDEM1-containing vesicles (EDEMosomes) and transported to the lysosome. Transmembrane insoluble glycoproteins could accumulate in the aggresome and then be degraded by the proteasome, or be targeted by an unknown mechanism to the lysosome for degradation.

If ER glycoproteins are not delivered to the Golgi compartment or degraded through ERAD, they become susceptible to aggregation. These aggregates are often insoluble in non-ionic detergent lysis buffers, such as 1% Triton X-100 (Kaganovich et al., 2008; Buchberger et al., 2010). Multiple mutant glycoproteins are detected as detergent-insoluble, and these aggregates are thought to play a role in the pathogenesis of numerous diseases (Rutishauser and Spiess, 2002; Kruse et al., 2006b; Fujita et al., 2007; Kroeger et al., 2009; Perlmutter, 2009), including neurodegenerative diseases and the liver disease associated with α1AT deficiency (described in more detail below) (Kaganovich et al., 2008). It is hypothesized that protein complexes or aggregates are too large to pass through the ERAD translocation pore, and hence must either be dissociated into monomers before ERAD, or degraded via a different process. Although the GQC system is clearly involved in targeting soluble ER glycoproteins to ERAD, it is not known whether GQC is involved in degradation of protein complexes or aggregates in the ER. Furthermore, studies are only beginning to elucidate the steps by which ER-derived glycoproteins accumulate in insoluble cytoplasmic aggregates (Fig. 4).

One example of a glycoprotein that accumulates in cytoplasmic aggregates is the cystic fibrosis transmembrane conductance regulator (CFTR), the glycoprotein whose mutant forms cause cystic fibrosis. CFTR is a large multipass integral membrane cyclic-AMP-dependent chloride transporter channel that undergoes inefficient folding and anterograde transport, and dislocates from the ER to form cytoplasmic, juxtanuclear aggresomes that are cleared by proteasomal degradation, as well as autophagy (Kopito, 2000; Farinha and Amaral, 2005; Fu and Sztul, 2009; Luciani et al., 2010).

In addition to membrane glycoproteins, luminal insoluble aggregates of ER glycoproteins can be transported out of the ER through a recently described vesicular system, distinct from COPII-coated vesicles used for ER-to-Golgi transport (Zuber et al., 2007; Le Fourn et al., 2013). These ER-derived vesicles contain high levels of EDEM1 and might be related to ‘EDEMosomes’, which are involved in degradation of ERAD components (a process termed ERAD tuning) (Bernasconi and Molinari, 2011). In a recent report detailing the degradation of glycosylated Aα-γ fibrinogen dimers, an elegant mechanism is described where these non-COPII vesicles bud from the ER and release the dimers into the cytoplasm, where they are then recognized by the selective autophagy cargo receptors p62 (SQSTM1) and NBR1, followed by packaging into autophagosomes and degradation (Le Fourn et al., 2013). The protein encoded by mutant α1AT Z allele, ATZ, which is associated with α1AT deficiency, also forms insoluble polymers in the ER that are eventually degraded by autophagy (Kruse et al., 2006a). The mechanism by which ER-localized insoluble ATZ is degraded by autophagy is not well understood, and perhaps the non-COPII-vesicle system plays a role (Fig. 4).

Insoluble forms of ER-entrapped proteins are often reported to accumulate in compartments that are in close association with the nucleus. These have been described by different investigators as ERQC compartments (also sometimes referred to as simply ERQCs) (Kamhi-Nesher et al., 2001; Kondratyev et al., 2007; Groisman et al., 2011) and ER-associated compartments (ERACs; which is the term we will use here) (Huyer et al., 2004; Fu and Sztul, 2009) based on their protein composition. ERACs have a juxtanuclear localization; some substrates are delivered and localized exclusively to these compartments and are not detected in other subfractions of the ER. ERACs are proposed to promote ER homeostasis by segregating aggregated (presumably non-functional, possibly toxic) proteins away from the functional ER (Benyair et al., 2011; Leitman et al., 2013) (Fig. 4).

The distinction between protein substrates that undergo degradation via ERAD or autophagy might not be as simple as suggested by the examples mentioned above, where soluble substrates are degraded by ERAD and insoluble substrates are degraded through autophagy. Indeed, CFTR has been demonstrated to both accumulate in cytoplasmic aggresomes and be degraded by HRD1-mediated ubiquitylation and subsequent degradation through the classical ERAD pathway (Johnston et al., 1998; Morito et al., 2008). It is very likely that a glycoprotein substrate with a propensity to misfold could be targeted to degradation by a combination of both the ERAD and autophagy pathways.

## Diseases of GQC

Either insufficient or overvigilant GQC can cause a wide range of pathological conditions. Below, we describe a select few conditions that have formulated our current view of GQC because they are used as models to decipher the GQC pathways. Congenital disorders of glycosylation (CDGs) illustrate the importance of glycosylation in the normal health and functioning of various human organs and tissues. α1AT deficiency demonstrates how mutations in a cellular protein overburden the GQC machinery, resulting in the accumulation of protein aggregates in the ER. Furthermore, experiments using wild-type and mutant alleles of α1AT have been instrumental in uncovering the surveillance and scrutiny that substrates undergo during every step of GQC. Much of our current understanding of the ERQC pathways is thus based on such studies. Finally, we describe the consequences of the loss of glycan-mediated trafficking in F5F8D. This discovery demonstrates how genetic studies of a disease can uncover physiological functions of components of ERQC pathways that have evolved to service an exclusive clientele.

### CDGs

CDGs are a set of rare human diseases that have very severe consequences and can always be traced back to either complete or partial loss of at least one of the components of the glycosylation pathway. Over 70 such disorders are now known, with abnormalities spanning almost every organ system (Freeze et al., 2012; Hennet, 2012; Freeze, 2013). The majority of these diseases are associated with mutations in the proteins of the N-glycosylation pathway. In particular, mutations in enzymes that catalyze the biosynthesis of the nucleotide sugars that form the substrates of N-glycan assembly as well as mutations affecting the enzymes that are responsible for the assembly of N-glycans are frequently observed. The convenience of using a simple diagnostic test that determines the N-glycosylation status of serum transferrin by isoelectric focusing is perhaps the reason for this over-representation of N-glycosylation pathway components in CDGs (Jaeken et al., 1984; Stibler and Jaeken, 1990; Hennet, 2012). With the advent of more sophisticated analytical methods, including next generation sequencing, consequences of the loss of more GQC components will very likely be discovered (Freeze, 2013). Recent reviews provide comprehensive analyses of various CDGs and their clinical manifestations (Freeze et al., 2012; Hennet, 2012; Freeze, 2013). Below, CDGs that are immediately relevant to GQC are highlighted.

TUSC3-CDG, MAGT1-CDG and DDOST-CDG result upon the loss of subunits in the OST complex, which catalyzes the en bloc transfer of the N-glycan core oligosaccharide onto the nascent polypeptide chain. Loss of TUSC3 or MAGT1 is known to cause intellectual disabilities (Garshasbi et al., 2008; Molinari et al., 2008; Garshasbi et al., 2011). Delayed psychomotor development (ability to walk) and failure to develop speech capacity were recently reported in a child lacking functional DDOST (Jones et al., 2012). GCS1-CDG (also known as CDG-IIb) arises when the affected individual lacks GS-I, the enzyme that cleaves the terminal glucose (Fig. 1; glucose ‘N’) from the N-glycan (De Praeter et al., 2000; Völker et al., 2002). The index case of GCS1-CDG was a compound heterozygote for two different GS-I missense mutations. Enzymatic analysis (of liver tissue and skin fibroblasts) demonstrated an almost complete lack of GS-I activity. The affected individual survived embryonic development but presented at about 2.5 months of age with multiple organ system failure, severe neurological defects (hypotonia, hypoventilation and seizures), dysmorphic features and progressive hepatomegaly (De Praeter et al., 2000). Although not yet categorized as a CDG, mutations in the β-subunit of GS-II had been previously linked with polycystic liver disease in humans (Drenth et al., 2003; Li et al., 2003; Janssen et al., 2010). Finally, MAN1B1-CDG is an autosomal recessive disease caused by the loss of ERMan I enzyme, and these patients also suffer from intellectual disability (Rafiq et al., 2011). Tissue-specific analyses have revealed that brain contains the largest N-glycoproteome (Zielinska et al., 2010), suggesting a potential connection to the frequent observation of intellectual disability as a consequence of the loss of N-glycosylation.

### α1AT deficiency

α1AT is a serine protease inhibitor synthesized primarily in hepatocytes, and is secreted into the bloodstream where it acts as the primary inhibitor of elastase in the lungs. α1AT deficiency is an autosomal codominant genetic condition resulting from any of the ~120 variant alleles described to date (Stoller and Aboussouan, 2012). The severity of the disease varies based on the allele, but the most common allele (found in nearly 95% of patients) is the Z variant (ATZ), characterized by a Glu342Lys missense mutation in the wild-type protein of 394 amino acids. This mutation renders the ATZ molecule inherently unstable with a propensity to self-associate, resulting in the accumulation of protein aggregates in the ER. Thus, the effect of the ATZ allele is twofold: (1) loss of functional α1AT in the bloodstream resulting in compromised connective tissue of the lung causing chronic emphysema; and (2) the accumulation of toxic aggregates in the hepatocyte ER, causing hepatitis, cirrhosis and an increased risk for hepatocellular carcinoma (HCC) (Valastyan and Lindquist, 2014). The extensive biochemical analysis of α1AT variants has proven indispensable in deciphering GQC and ERAD pathways. In addition to the common disease allele ATZ, many of these studies have employed the null Hong Kong (NHK) allele that results in a truncated form of α1AT (Sifers et al., 1988). In turn, this clarified picture of α1AT folding in the ER promises to introduce more effective therapeutic approaches for patients (Bouchecareilh et al., 2010; Marciniak and Lomas, 2010).

Like all glycoproteins, the folding status of α1AT is appraised at every stage of its biosynthesis by a host of proteins inside the ER, and is guided through the folding, export and ERAD pathways, as appropriate. α1AT undergoes N-glycosylation at three distinct sites during its biosynthesis. Immediately after the terminal glucose residue is removed by GS-I, α1AT associates with malectin, which specifically recognizes the Glc_2_Man_9_GlcNAc_2_ form of the N-glycan. The function of malectin binding to the nascent glycopeptides was studied using wild-type and NHK alleles of α1AT (Chen et al., 2011; Galli et al., 2011). Malectin was found to stably associate with NHK, but not wild-type or unglycosylated NHK, and limit NHK secretion. Furthermore, decreased secretion of NHK upon malectin overexpression was found to be a consequence of enhanced degradation of luminal NHK in an OS-9-dependent manner (Chen et al., 2011). Given that every nascent glycopolypeptide undergoes GS-I-mediated deglucosylation, how does malectin selectively withhold misfolded NHK? Qin et al. discovered that malectin interacts with ribophorin I, a subunit of the OST complex that has chaperone-like activity (Qin et al., 2012). It is likely that malectin binds to Glc_2_Man_9_GlcNAc_2_ glycans of all glycoproteins but retains only the misfolded molecules in a stable ternary complex with ribophorin I. Whether malectin itself has peptide-binding activity remains to be investigated.

Misfolded α1AT that escapes the malectin GQC checkpoint enters the CNX cycle after glucose trimming by GS-II. Indeed, Galli et al. demonstrated that malectin does not compete with CNX for substrate binding (Galli et al., 2011). Similarly to malectin and Glc_2_Man_9_GlcNAc_2_ glycoproteins, the ER lectins CNX and CRT bind to incompletely folded or misfolded α1AT in the ER; however, they do so via Glc_1_Man_9_GlcNAc_2_ glycans (Ou et al., 1993; Le et al., 1994; Wu et al., 1994). Immediately after release of the substrate from CNX/CRT, GS-II removes the lone remaining glucose from the N-glycans, allowing interrogation by UGGT1 (Choudhury et al., 1997). Importantly, reglucosylation of NHK and ATZ molecules by UGGT1 directs their re-entry into the CNX cycle and improves their solubility (Ferris et al., 2013). Interestingly, the consequence of this increased solubility differs depending on the mutant; whereas UGGT1 improved secretion of the non-native form of NHK, it reduced aggregation of ATZ and retained it in the ER in a soluble, misfolded form (Ferris et al., 2013). These findings indicate that even modulation of a single component of GQC can have completely different outcomes based on the particular mutation in the substrate.

Studies using α1AT alleles have helped formulate our current understanding of glycoprotein export and ERAD pathways. As substrate proteins exit the CNX cycle, misfolded candidates are demannosylated and targeted for degradation. The transmembrane region of CNX associates with EDEM1 and targets misfolded NHK molecules to the ERAD pathway (Oda et al., 2003). ERMan I, the principal candidate that primes substrate proteins for degradation, was characterized using NHK as a model (Cabral et al., 2000; Cabral et al., 2002; Wu et al., 2003; Karaveg and Moremen, 2005; Karaveg et al., 2005; Hosokawa et al., 2007; Avezov et al., 2008). For instance, degradation of luminal misfolded NHK was significantly hindered upon abrogation of ERMan I activity either by treating with the inhibitor kifunensine or by siRNA-mediated knockdown. The fraction of NHK that escaped scrutiny in the ER or entered the Golgi via bulk-flow was demannosylated by the resident Golgi Man IA, IB or IC enzymes and returned to the ER for degradation (Hosokawa et al., 2007). Finally, direct interaction of NHK with the ERAD lectins OS-9 and XTP3-B mediates delivery of misfolded NHK to the HRD1-SEL1L complex for degradation (Bernasconi et al., 2008; Christianson et al., 2008; Hosokawa et al., 2008; Hosokawa et al., 2009; Mikami et al., 2010). A recent study, however, indicates that XTP3-B can bind Man_9_GlcNAc_2_-NHK and inhibit its degradation (Fujimori et al., 2013). Nevertheless, these studies on α1AT have helped define the mechanisms of multiple back-up systems to ensure tight surveillance in GQC.

### Combined deficiency of F5 and F8 (F5F8D)

Coagulation factors V and VIII (F5, F8) are homologous glycoproteins with a conserved domain organization. They require complex folding and post-translational processing to attain their final functional structures and be secreted. Both proteins are extensively glycosylated in the ER and handled by the GQC machinery before being exported to the Golgi. F5F8D is an autosomal recessive disorder distinct from hemophilia A (F8 deficiency) and parahemophilia (F5 deficiency) in that the genetic loci of F5 and F8 remain unperturbed. The molecular basis of F5F8D remained a mystery until forward genetic analyses uncovered mutations in the *LMAN1* and *MCFD2* genes (Nichols et al., 1998; Zhang et al., 2003). Lectin mannose-binding protein 1 (LMAN1) is a type I transmembrane protein originally identified as ERGIC-53, a 53 kDa protein of unclear function that localizes to the ER-Golgi intermediate compartment (ERGIC). MCFD2 is a soluble 16 kDa EF-hand protein that associates in a Ca^2+^-dependent manner with LMAN1 to form the cargo receptor that cycles between the ER and ERGIC. Both LMAN1 and MCFD2 directly bind to both F5 and F8, LMAN1 through its lectin-like carbohydrate recognition domain and MCFD2 through its EF-hand domains, and deliver F5 and F8 to the ERGIC (Zhang et al., 2005). The normal levels of other plasma proteins in individuals with F5F8D suggests that LMAN1 and MCFD2 might have evolved to selectively assist trafficking of only F5 and F8. Thus, genetic analyses of F5F8D patients clarified two important aspects of protein folding and trafficking: (1) the role of LMAN1 as well as its partner, MCFD2, in the ER-to-Golgi trafficking pathway; and (2) that the efficient export of F5 and F8 requires specialized machinery for selective cargo, revising the presumed bulk-flow model.

Intriguingly, LMAN1 and MCFD2 are expressed in lower organisms that do not express either F5 or F8, leaving the possibility that they are involved in servicing additional proteins. Indeed, *in vitro* experiments using cell-culture systems have demonstrated that cathepsin C (Vollenweider et al., 1998), cathepsin Z (Appenzeller et al., 1999; Appenzeller-Herzog et al., 2004; Appenzeller-Herzog et al., 2005) and α1AT (Nyfeler et al., 2008; Zhang et al., 2011) are cargos for the LMAN1-MCFD2 complex. In contrast, mice deficient in LMAN1 do not display any discernible defect in the hepatic intracellular levels of cathepsins Z and C, or plasma levels of α1AT, although they do accumulate α1AT in the liver (Zhang et al., 2011). It is possible that, unlike other substrates studied so far, F5 and F8 proteins need to be exclusively serviced by the LMAN1-MCFD2 complex.

## Model systems to study GQC

Studies on pathological conditions resulting from abnormal folding and/or quality control of cellular proteins facilitated the discovery of multiple ERQC pathways and their significance in cellular proteostasis and organismal health. The mechanistic intricacies of these pathways, however, have required cellular and animal models that could be manipulated in more defined scenarios. Thus, almost every component of the GQC has been systematically knocked down or ectopically expressed, followed by interrogation using model glycoproteins to study their specific functions in a cellular context. Additionally, animal models in which genes of the ERQC pathway are disrupted have proven invaluable in understanding the effects of such mutations at the physiological level. Indeed, several of these proteins are essential for embryonic development and survival, making studies on such gene mutations in humans impossible. Here, we briefly discuss a number of knockout mouse models related to GQC and important findings produced from their study.

*Crt* deletion in mice results in embryonic lethality due to irrecoverable disruption of Ca^2+^ homeostasis during cardiac development (Mesaeli et al., 1999; Guo et al., 2002). The mechanism for *Crt^−/−^* embryonic lethality likely involves calcineurin activation and nuclear translocation of MEF2c (Lynch et al., 2005). *Cnx* deletion, by contrast, results in postnatal lethality, with half of the *Cnx^−/−^* animals dying 48 hours after birth. Surviving *Cnx^−/−^* mice have motor problems with decreased myelination of nerve fibers (Denzel et al., 2002). The dramatic phenotypes observed for *Cnx* and *Crt* deletions demonstrate the absolute requirement of these chaperones for mammalian survival, and suggest that, although homologous in structure and function in the GQC system, these proteins play distinctly vital roles that cannot be compensated for by the other. At the cellular level, deletion of *Cnx* and/or *Crt* was demonstrated to affect multiple aspects of protein folding, including the folding rate, efficiency and fidelity of model substrates (Molinari et al., 2004). Specifically, studies utilizing *Crt^−/−^* cells demonstrated a role for CRT in ensuring optimal assembly and antigen presentation by the MHC class I molecules (Gao et al., 2002; Howarth et al., 2004; Howe et al., 2009).

Embryonic lethality was also observed upon deletion of three other components of GQC: *Grp78* (*Hspa5*; which encodes BiP)-deleted embryos die as early as E3.5 (Luo et al., 2006), *Uggt1*-null embryos die at E10.5 (Molinari et al., 2005) and *ERp57* (*Pdia3*)-null embryos die at E13.5 (Coe et al., 2010). However, ablation of ERp57 specifically in B cells demonstrated its role in the process of antigen presentation; ERp57 is dispensable for the oxidative folding of substrates but is required for recruitment of MHC class I molecules to the antigenic peptide loading complex (Garbi et al., 2006).

Upon discovering that mutations in the *LMAN1* and *MCFD2* genes lead to F5F8D, these genes were deleted in mice to further study the mechanism of F5F8D using a reverse genetics approach. As expected, plasma levels of F5 and F8 in the *Lman1^−/−^* animals decreased, to half of the normal levels (Zhang et al., 2011). Despite the absence of any apparent defects in the production of other plasma proteins or COPII-coated-vesicle formation, *Lman1^−/−^* hepatocytes displayed slightly distended ER with significant accumulation of α1AT and BiP, suggesting a disruption in proteostasis (Zhang et al., 2011).

Finally, transgenic models expressing mutant glycoproteins have also proved very useful in deciphering the GQC pathways and their role in the pathophysiology of human disorders. Thus, mice expressing human ATZ helped demonstrate the role of ER-localized accumulation of misfolded ATZ in causing hepatic fibrosis. Pharmacological activation of autophagy with carbamazepine partially ameliorated the fibrosis, and this might represent a potential therapeutic avenue to treat the liver pathology of α1AT deficiency (Hidvegi et al., 2010).

## Conclusion and outlook

Protein folding is the most error-prone step in gene expression. As a consequence, cells have evolved sophisticated surveillance systems to limit protein misfolding and to eliminate irreversibly misfolded proteins. N-linked glycosylation of proteins in the ER initiates entry into a complex glycoprotein-specific quality-control system (GQC) that maintains the fidelity of the secretory and transmembrane proteome. GQC is quite sensitive, recognizing glycoproteins with even slight amino acid substitutions, and targeting them for ERAD (despite their potential to contribute a measure of bioactivity). The CNX cycle features prominently in GQC, with the reglucosylating enzyme UGGT1 serving as a primary folding sensor that dictates downstream decisions regarding folding, trafficking and degradation. How the GQC system functions to degrade terminally misfolded glycoproteins, and what happens when these degradation pathways are overwhelmed, are key questions for future exploration. Although many of the components of GQC have been identified, recent discoveries related to the targeting of misfolded proteins to the ERAD by malectin (Galli et al., 2011) and O-mannosylation of unfolded proteins (Xu et al., 2013) makes it likely that additional GQC components remain to be identified. The discovery of LMAN1 and MCFD2 illustrates how specialized systems have evolved to address folding and export of a limited set of related proteins. It also serves as a reminder of the complexity of ER-folding, quality-control and export pathways, for which, despite the strides made by the scientific community of late, many exciting discoveries lie ahead. Greater understanding of the mechanistic details of GQC will help identify potential therapeutic targets and inform the pathogenesis of the ever-increasing list of ER storage diseases.
